# Nothing about us without us! How users configure clinical treatment in Italian residential communities: ethnography of therapeutic engagement

**DOI:** 10.3389/fpubh.2024.1460985

**Published:** 2024-12-03

**Authors:** Antonio Iudici, Tobia Berardelli, Davide Fenini, Jessica Neri, Emiliano Subissi

**Affiliations:** ^1^Department of Philosophy, Sociology, Education and Applied Psychology (FISPPA), University of Padua, Padua, Italy; ^2^Institute of Psychology and Psychotherapy, Scuola Interazionista, Padua, Italy

**Keywords:** drug users, drug community, health, treatment, qualitative research, ethnography

## Abstract

The effectiveness of the interventions in the Therapeutic communities (TC) depends not only on the quality of the specialized knowledge and methodologies adopted, but also on the meanings consumers give to them. Building the therapeutic alliance is a key element in reducing high drop-out rates and predicting more favorable outcomes. This research investigated the discourses practiced by 45 people with substance use disorders who had been accessing a therapeutic community for less than 15 days, with the aim of delving into the meanings given to treatment and pathway goals in the service, to analyze what theories are used to explain consumption and therapeutic change. The study made use of Discourse analysis (DA) and Positional theory with a protocol of written open-ended questions. The results show how participants configure the community pathway adopting a passive role, underestimating the need to co-participate in treatment design and bringing in poorly formalized goals. The collected texts provide a better understanding of the perspective of community users, emphasizing the need to share spaces of co-design from the very beginning in order to promote empowering, reasoning about the implications of the various discourses produced by consumers to explain their autobiography and to envision paths of change.

## 1 Introduction

A therapeutic community for people who use substances (TC) is a residential service whose aim is to help people reduce and eliminate the use of psychotropic substances and reintegrate into society (1). They are mainly based on two assumptions. The first concerns the fact that treatment is of the person as a whole, not specifically on the basis of substance use (2). In a TC, rather than classifying individuals according to their drug abuse patterns, they are instead delineated according to degrees of ‘psychological dysfunction’ and ‘social deficits’, ‘existential difficulties’ (3). Substance use is considered to be a problem that affects the whole person and the way they interact with their life context. Cognitive, behavioral, existential problems (4), mood disorders (5) are also often associated with substance abuse. Frequently there is a crash of values that are often confused, antisocial or moral (6, 7). Essentially, addiction can be seen as a symptom rather than the essence of the disorder (8). The second relates to the fact that the pathway of change or treatment occurs through the involvement of a community or group (9). In CT, an attempt is made to break the link with the substance and to direct the person who uses psychotropic substances toward a rehabilitation of his or her life context. Moreover, many of these people often come from a socially depressed sector, where the customs and values of society are ignored or totally shunned. From a CT perspective, a change in the person’s lifestyle and personal and professional identity is considered crucial for recovery to occur.

These characteristics explain the differences between the CT model and standard inpatient psychological and psychiatric treatment, which is much more based on symptom-oriented diagnostic systems such as DSM5 and ICD (3). It is important to say that therapeutic communities, like any other service, are not a one-size-fits-all solution for all people who use substances. Rather, it is often collaboration between services that makes treatment effective. For some patients, the medicalisation of addiction has enabled the integration of pharmacological and specialized approaches, for example by integrating action and behavioral interventions with medication-assisted treatment (MAT) (10, 11). For others, medicalisation has however fostered a progressive overlap between users and patients, inappropriately applying and generalizing the idea of illness to people who use substances (12). Interventions and research have often focused on neurophysiological and behavioral aspects (13), neglecting the perspectives of people accessing services, their goals and the meanings attributed to treatment and consumption (14–16). Indeed, this work responds to the need to complement substance use studies in quantitative terms (17, 18), with qualitative studies that can better access users’ experiences from their perspective (19, 20), hence the context of therapeutic communities, within which our research takes place. The literature has highlighted how the phase of access to CT can be decisive in the course of the programme (17, 21), favoring or hindering the construction of the therapeutic alliance and orienting the pathway toward shared goals (22–24). Several studies point out that one of the reasons for therapeutic failure among people seeking treatment for substance use is the therapeutic alliance (25–27) and that the first month is decisive for continuation (28, 29).

However, this access or reception phase is often seen as external and prior to treatment and therefore poorly attended to, with the result that most consumers access treatments about which they have little information, playing a passive role and little involvement in the planning stages (30–33).

This is also fostered by the social prejudice that sees people who use substances as lacking the ability to self-determine and actively contribute to choices regarding their pathway (34, 35). The lack of co-design dynamics contributes to generating the situation whereby many users decide to prematurely discontinue programmes and migrate to other facilities deemed more in line with their demands, on the basis of elements that are then systematically disregarded leading to the abandonment of the idea that an intervention can be effective (36). A better involvement of users in the CT would overcome a dehumanizing and objectifying view of the people seeking treatment for substance use (37, 38), whose relationship with CT staff is often built on a rigid ‘us versus you’ approach that relegates professionals to a controlling role that severely undermines the therapeutic potential of services (25). This is also in light of another literature finding that the effectiveness of a therapeutic community (TC) programme is directly related to the amount of time spent in the service (17, 39, 40). It is, moreover, well known that it is important to build a dialogue with the user from the beginning of the pathways (41–43), to introduce elements of flexibility that allow the person to feel an active part of his or her pathway and that make the premises on which the treatment is based shareable, favouring a process of widespread empowerment between staff and user (44–46). Studies have shown that treatment can reduce dropout when the user’s family is also involved (47). It has also been found that one of the aspects considered most relevant by users when assessing the quality of a programme is the communication competence and helpfulness of the staff, which can be generalized to the perception that they are important in shaping their own treatment (48).

High drop-out rates within CTs are therefore particularly critical to their functioning (49, 50), and these are often attributed to the patient’s own pathologies or abnormalities rather than promoting reflection on the access and treatment modalities offered in services (26).

This research delved into the meanings attributed by people who use substance accessing a TC regarding treatment and its goals, to analyze the discursive constructions from which the interaction between user and service takes shape from the earliest stages of their stay in the service. Formulated from a personal conceptualization of consumption and one’s own issues, participants’ responses allowed for the investigation of a number of questions: what goals do consumers intend to achieve through a TC pathway? How should treatment take place? What should be the role of TC? What will be changed once the stay in the service is over? From the collected texts, it is possible to analyze how discourses about drugs available culturally and within services provide a vocabulary from which events in one’s life are read: what metaphors are adopted to explain consumption? Is the goal to change, to heal, to acquire something, or to remove a negative? The research thus aims to provide a better understanding of users’ experiences and the elements considered salient for change, as well as the strategies contemplated and the meanings attributed to them, in order to structure new ways of designing and sharing what happens in a TC so as to increase users’ compliance and satisfaction, and thus the effectiveness of interventions.

## 2 Method

### 2.1 Theoretical background

The study utilizes the interactionist paradigm as its theoretical foundation (51–55). Within this paradigm, the interactive process is considered fundamental to the construction of reality. Individuals, in this case people who use substances, are seen as continuously engaged in negotiating and shaping their reality through the attribution and use of meanings, guided by specific socio-cultural frameworks (56–59).

In this perspective, self-representation and identity, as a dialogic and idiosyncratic process, may change according to the different contexts experienced by the person using substances (60–62). Through this approach, the therapeutic community stimulates the user to have experiences that may allow a new self-concept and a shift in identity toward socialized roles that do not involve consumption (63).

Within the individualized projects, therapeutic activities are thus chosen according to (and with) the person who uses substances and organized with the aim of creating new existential possibilities and new self-narratives.

### 2.2 Aims

The aim of the research is to investigate how individuals who consume legal and illegal psychoactive substances shape the service and treatment during the entry phase into a TC. Specifically, considering the potential biographical transition represented by entering such a service, attention has been focused on the main area of investigation: the discursive configuration regarding the treatment.

Focusing on this area allows for a deeper understanding of the discursive processes used to shape the treatment and intervention programs proposed by the residential community, and to tailor them based on the users’ objectives and expectations.

**Figure 1:**
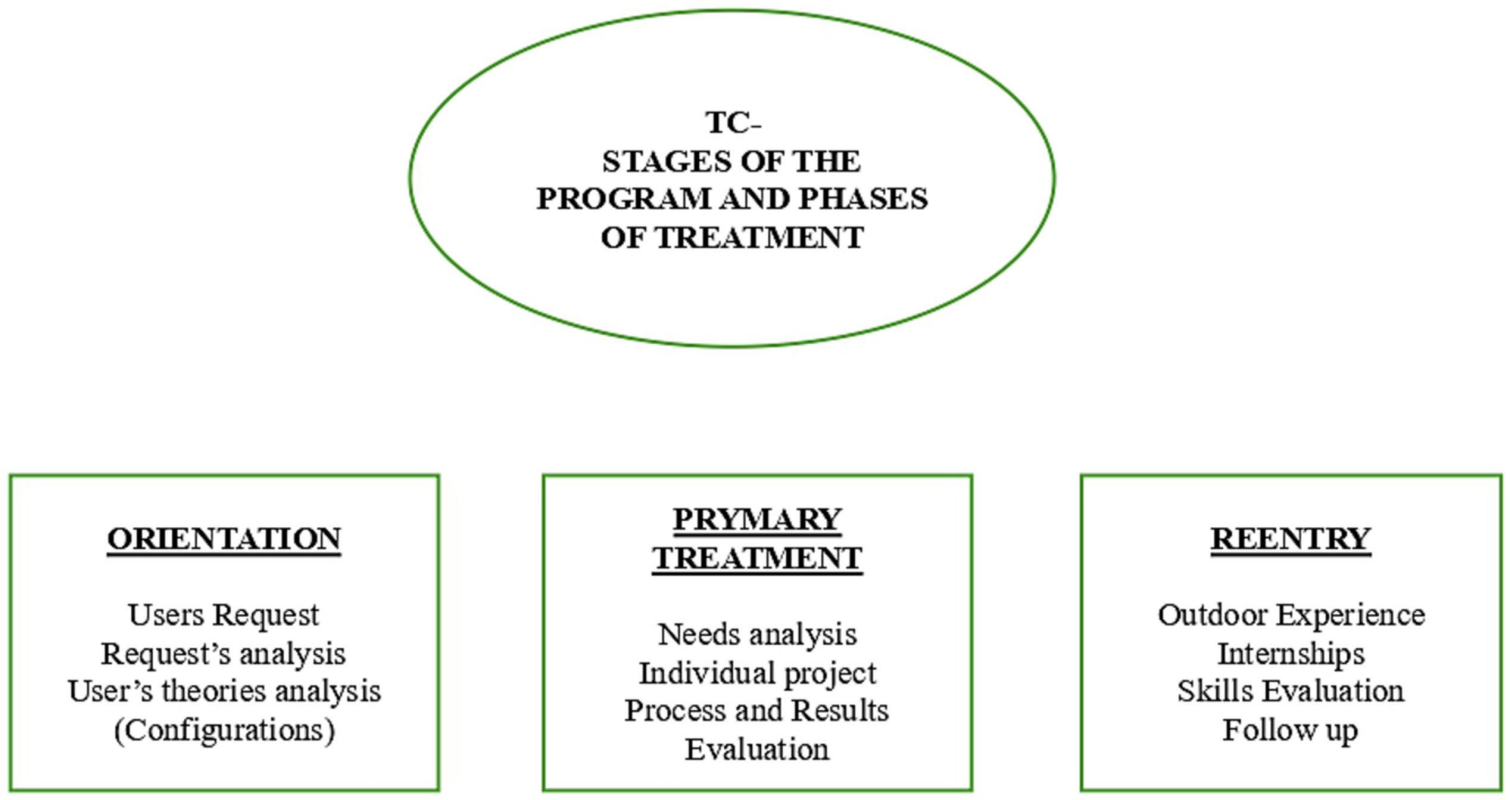
Phases of treatment conceptual diagram.

This area of investigation allows for an in-depth exploration of the theories, beliefs, and expectations of service users regarding the entry and utilization of interventions promoted within these contexts (Figure 1).

### 2.3 Participants

The research was conducted in Italy. The study involved 45 individuals who had accessed four therapeutic-rehabilitative residential services for drug addiction within the past 15 days. To recruit potential participants, a mapping of local services in these regions was performed, focusing on those offering programs for substance-related issues management and treatment. The identified facilities were then contacted, and the research objectives were communicated and shared to the relevant staff.

Participants were selected based on their recent admission to the residential service. These individuals were approached and invited to participate, sharing research objectives and methods. Each participant was provided with a form to give their informed consent and was asked to complete a written questionnaire. The research team remained available to clarify any doubts and encourage participation.

The study received approval from the University of Padua’s ethics committee for research. The demographic data of the participants are summarized in Table 1.

**Table 1:** Demographic data participants.

		*N*	%
Gender			
Male	34	75.5
Women	11	24.5
Age range			
18–30	15	33.3
31–50	22	48.9
51–70	8	17.8
Age average	35.34	/
Typology of entry			
First entry	30	66.67
Alternative measure program	15	33.33

Notably, the majority of participants are male, with an average age of 35.34 years, which aligns with the national average reported by the Department of Anti-Drug Policies (64).

### 2.4 Data collection

Qualitative methods were prioritized to elucidate the theories, beliefs, and expectations of participants and service users concerning the initiation and utilization of interventions within these contexts. Analyzing narratives enabled the identification of individual and interpersonal actions, meanings, and social processes that shape interpersonal relationships, even within institutional settings (65, 66).

The research is conceptualized as a relational, collaborative practice and dialogical process (14, 67), wherein the object of analysis is co-constructed by both researchers and participants.

The research instrument comprised a written protocol consisting of four open-ended questions (see Table 2), designed around the investigation’s focus: the discursive configuration regarding the development of the intervention.

**Table 2:** Research protocol.

Research area: Discursive configuration regarding the development of the intervention
1. How do you envision the treatment process unfolding in a TC?
2. What goals do you intend to achieve in this process?
3. How do you think the TC could facilitate you in achieving your goals?
4. Imagine yourself at the end of the treatment with the TC you are in. What do you think will have changed compared to today?

Attention was dedicated to analyzing the discursive processes shaping treatment and intervention programs proposed by the residential community, adapting them according to users’ objectives and expectations.

The questions were carefully formulated to stimulate the production of text relevant to specific research issues while allowing for diverse content and forms (68).

The selection of open-ended questions was crucial within the qualitative framework (65, 66), especially in the context of written text collection (69–71). The prompts were tailored to the research objectives and specific areas of inquiry to delineate potential discursive scopes. Simultaneously, their open-ended nature afforded participants ample freedom to express themselves and manage their responses and rapport with the researcher (Table 2).

The questions were presented to participants individually, providing clarifications about the methods and objectives of the research, ensuring anonymity and assurance that the produced texts would not be disclosed to service personnel. The researcher was available to clarify any questions during the completion process. Responses were provided in written form autonomously, taking between 25 and 60 min.

The responses were transcribed verbatim and, before delivery, were reviewed with the participant to ensure understanding of the written text (72, 73). The completion occurred within 15 days of entering the community.

### 2.5 Data analysis

The approach of Discourse Analysis (74, 75) was employed to analyze the texts. This method involves a systematic examination of language use to understand how discourse constructs and shapes social realities, identities, and interpersonal dynamics and processes. This method goes beyond mere content analysis by focusing on the linguistic structures, rhetorical devices, and discursive strategies employed in texts. Discourse Analysis aims to uncover the underlying meanings and ideologies embedded within language. It explores how language is used to construct specific versions of reality and to influence perceptions, beliefs, and actions. This approach is particularly attentive to how discourse reflects and perpetuates social norms, cultural values, and institutional practices.

Central to this method is the recognition that language plays an active role in constructing and maintaining social reality and specific discursive configurations.

The framework of Positioning, developed by Davies and Harré (76), Harré and Van Langenhove (77), and Harré and Moghaddam (78), is a conceptual tool within Discourse Analysis that focuses on how individuals and social actors are positioned or position themselves within discursive contexts.

The conceptual tool of Positioning (76–78) was utilized to underscore the dynamic aspects through which the discursive shaping of identity occurs, including that of the consumer and user within the community context. Positioning theory asserts that individuals construct their identities and make sense of their social worlds through the positions they adopt or are assigned within conversations and interactions. These positions are not fixed but are dynamically negotiated through language and discourse. They reflect and shape individuals’ relationships, roles, and identities within specific social settings (76).

Within the context of Discourse Analysis, positioning involves identifying and analyzing the ways in which language is used to position individuals and groups within social hierarchies, roles, and identities. This framework examines how discursive practices allocate rights, responsibilities, and authority to different participants in conversations or narratives (79).

Moreover, positioning theory highlights the performative aspect of language, where speakers use discourse not only to describe but also to enact and reinforce social positions and relationships. It emphasizes the role of language in constructing social reality and influencing the distribution of power and authority among participants.

The analysis paid special attention to socio-cultural and ethnopsychological elements of the narratives, recognizing their significance in the discursive construction of the investigated reality. This comprehensive approach illuminated how text and discursive practices not only reflect but actively construct social realities and identities, coherently with the research aim (62, 80, 81).

Through the analysis and positioning, we were able to explore how participants articulate their narratives and positions regarding the area of investigation: the discursive configuration related to the development of interventions. The details of these configurations encompass the discursive processes that shape the treatment and intervention programs proposed by the residential community, as well as how they shape and adapt users’ objectives and expectations.

Analyzing discursive processes allows for the identification of relevant discourses and positions, thereby elucidating their practical implications within participants’ life stories and intervention pathways.

The analytical process involved several distinctive steps:

- A comprehensive examination of texts generated by participants, focusing on the research area and its discursive nuances;
- Evaluation within the research team to review the preliminary text analysis, identifying and resolving any discrepancies;
- Definition of fundamental discursive positions aligned with the research area and objectives;
- Grouping of findings into key positionings to enhance clarity and facilitate interpretation of the results.

These delineated positionings and their articulations, consistent with our analytical approach, are recognized as discursive processes that critically influence the emergence of the phenomenon under investigation (82).

## 3 Results and discussion

The responses collected through the protocols make it possible to analyze the configuration within which the citizens accessing the services represent the program and treatment in Residential Community. It can be grouped within five categories that we have named: treatment as detoxification, treatment as psychological intervention, treatment as skill learning and treatment as pursuit of an ideal life. Let see below how these categories are characterized.

### 3.1 Treatment as detoxification

For a small proportion of users, treatment coincides with the detoxification process; within this configuration, the goals are the withdrawal of drug therapy, generally substitution therapy, and the overcoming of the state of biochemical dependence. This is often found in phrases such as: *‘I am here to get off drugs’* (P33), *‘I will stay here until I get off methadone’*, (P32) *‘I have to get help until I no longer touch drugs’* (P24).

One participant summarizes the effectiveness of treatment by saying: *‘Every day it is important to have one more day of abstinence from substances’* (P6); indeed, within this configuration, maintaining abstinence is both a goal and an outcome. The social and physical isolation resulting from the residential nature of the service is considered as strategic to support the processes of release from consumption. In this perspective, some participants theorize the difficulties of progressive pharmacological scaling up and anticipate the longitudinal increase in the desire to consume, also foreshadowing some of the difficulties of the pathway. Such theorisations take on the sense of a script that arranges in the pathway some *“difficult moments”* (P38) and *“you have to go through a long period of abstinence”* (P31).

In this respect, one participant says: *‘The first months are the most difficult, then you slowly get into the spirit of it and you get better and better with each passing day’* (P14).

Another states: *‘the support of the group will be important, fundamental in everyday life and in moments of discouragement’* (P16). Another respondent states: *‘the TC will have to help me in the withdrawal period, which I do not know how strong it can be’* (P26).

The focus of the users within this configuration is to resist and improve, there seems to be no other viable discourse concerning life or personal plans.

### 3.2 Treatment as psychological intervention

A second category of responses defines treatment as a psychological intervention; it was noted that people trace the core of their discomforts back to emotional and cognitive issues, using a psychologising vocabulary that implies treatment interventions of a predominantly psychological nature. Participants often use expressions such as ‘trauma’, ‘illness’, ‘disorder’, ‘demons’ and ‘dark sides’ to talk about the consumption of psychotropic substances, without reference to the contexts they belong to and the dynamics through which these elements have progressively consolidated consumption.

In this regard, one participant says: *‘Overcoming the traumas that led me to the use of alcohol and substances’* (P7), implicitly reiterating a positioning as a powerless person. In this case, one’s agency fails not so much in the face of the substance as of the ‘trauma’, which is also described as a reality independent of oneself.

The treatment is therefore configured as purely psychological and winds in two directions: on the one hand the search for causes that explain the consumption or its recurrence, and on the other the resolution of the problem (problem solving).

With regard to the search for the psychological cause, for example, some participants say*: ‘I have to understand the cause of certain thoughts’* (P30), or *‘It is important for me to understand what leads me to make the same mistakes over and over again*’ (P31), *‘And to try to understand from an expert what in the past led me to consume substances’* (P35). Such formulations are used to position oneself at the mercy of internal forces and thus as passive receptors of a process of unraveling and clarification, understanding and healing. The tendency is to reify a psychological problem, then considering it to be the cause of behavior (83), from which processes of de-empowerment ensue.

The dimension of persuasion is well exemplified by one participant, who states: *‘TC has to make me understand that I should not touch alcohol anymore’* (P16). Understanding one’s own conduct returns as a central element with another participant saying: *‘The one and only thing I want and I do not think it will be realised is to understand definitively that at my age I cannot afford to go back and waste any more time…sure that the negative consequences will get worse if I do not change’* (P10).

Another participant says: *‘Comparing myself with my peers and understanding the cause and effect of certain actions in order to understand how I can cope on my own’* (P32).

Regarding the resolution of the problem, also referred to as ‘trauma’, ‘obsessive disorder’, ‘dark side’, discourses are used to find ways to make the consumption stop.

One participant says: *‘I want to get rid of the obsessive desire for substances’* (P1), while another says: *‘At the end of the path I hope to have conquered my demons’* (P3).

The resolution of the problem also transits through the regaining of a sense of self as a person of value, reported by participants as ‘self-confidence’. ‘*I have to sustain my path thanks to psychotherapy, pharmacology… but above all by believing in me’* (P7), *‘After this path, I hope my ex-partner can regain confidence in me, and I hope I have also regained it’* (P1).

One participant says: *‘I want to be able to get out of TC and be independent, believe in myself more and no longer rely totally on the person next to me’* (P8).

### 3.3 Treatment as learning personal skills

A third category of responses configures treatment as learning skills, within this representation the user does not have a passive role of delegating the solution of the problem to experts, but introduces an developmental and progressive dimension of the intervention through the use of the metaphor of the “gym” where to implement new skills in order to ‘come out of here as a stronger person.’

One participant says: *‘It’s like a gym where you train to be strong in the daily routine of life, which often trips you up. It is easy for us addicts to fall in front of frustrations, pain, stress, and take refuge there, in the comfort zone we know. TC trains you to find other strategies to overcome the bad moments’* (P17).

Another participant says: *‘At the moment I cannot see the end of the treatment, but I hope to come out of here as a stronger person to manage my emotions without substance use’* (P7), another says he wants to *‘Learn not to be conditioned by bad company. To prove with facts that I am as strong as I think I am and finally win at everything’* (P11). In some cases, the intention to learn to deal with problems and to have to become strong and resilient is based on a view of oneself as incapable or as a weak person.

In relation to his own weakness, one participant states that he wants to *‘Eliminate methadone use and get to have an armour that protects me from thoughts and events that can trigger the need and craving for opioids in me’* (P26). The term ‘armour’ is illustrative of how the need to strengthen oneself is interpreted, related to the strength needed to cope with substance use.

To experiment and harden oneself, the possibility of living a plurality of experiences (inside and outside the service) is valued, and the TC is seen as a safe and protected space in which to test oneself. Value is placed on the repetitive aspects of the TC days, stating that *‘rules fortify’* (P32).

One participant claims that *‘Each course is strictly personal, but the organisation of time is very important…time to eat, time to work, groups, activities’* (P8).

For some, respecting these rules can foster the discipline needed to carry out certain activities of everyday life, as well as being an opportunity to learn how to organize time and the more mundane aspects of daily life; indeed, one participant says: *‘Basically everything we do…the sharing together, the sharing of tasks - breakfast, cleaning, washing…everything serves a single purpose’ (P6), while another reports: ‘I want to learn not to think about drugs to fight boredom’* (P21).

In general, the everydayness of TC, exemplified by the mantra of ‘living day by day’, is seen by the participants as a functional strategy for a search for ‘independence’, ‘autonomy’ and ‘responsibility’ in which, for many, the primary goal is realized.

### 3.4 Treatment as an overall life change

In this fourth category, treatment is represented as an opportunity to redesign one’s life as a whole. One participant states *‘By the end of the course I hope everything will have changed’* (P14).

The goal becomes a radical transformation of the self, sometimes with an ideal connotation, in order to assume the status of a ‘real person’, otherwise crushed on consumer aspects alone.

A participant asserts: *‘I have to get to be a real man, not just an alcohol addict’* (P13), and another says: *‘At the end of treatment I imagine myself as a mature man, able to cope and make good decisions’* (P15).

Recurring themes relate to medical, housing, work, bureaucratic issues, indicators concerning the need for a new socialization.

For some participants these goals are conceived as desirable consequences of a personal transformation, but others see getting a job and a house as the only criteria for assessing the effectiveness of the service.

We find some examples in the following responses *‘I hope to get a decent life, without needing to ask the priest for the 10 euros…to have a meagre job according to my abilities’* (P4), another participant states: *‘Getting a driving licence - finding my own flat - gaining knowledge in the world of work - fixing my teeth’* (P10), while P11 reports: *‘A change of lifestyle…changing acquaintances and getting out of the circle that led me to substance use’*, and another says: *‘The TC can help me by finding me a job, if I behave well’* (P19).

The ‘neutral space’ of the TC makes it possible to think about a series of hitherto neglected or compromised aspects, which contribute to flesh out what, for many, seem to be essential elements for a ‘decent’, ‘healthy’, ‘normal’ life.

Treatment is also identified as an instrument of social redemption, with practices devoted to making oneself seen as different from the people who are significant to them, for example, friends and family. We find examples of this representation in the following answers: *‘The goal is to regain my family, to stay with my children, my wife and to return to being a present father and husband in every moment of the day’* (P14), *‘It is fundamental that next to you walk your loved ones, and if possible that they also ask for help…. the TC is your own space, where you choose the relationships to continue and those to interrupt and where you can “heal” thanks to the help of the operators who can act as mediators with reference to the healthy relationships you want to recover’* (P16), *‘My goal is to resume the relationship with my father and my brother who have not spoken to me for a long time’* (P17).

## 4 Discussion

Some of the participants report an idea of TC as detoxification and pharmacological management, the request is exhausted in ‘not using substances’, the goal is abstinence. The interviewed users are thus positioned as carriers of a biological addiction, from which arises a request for help of a charitable nature that is exhausted in the divestment of substances and drugs, binding them to poorly negotiable and rigid goals.

Configuring treatment exclusively on substance use leads to maintain the idea that the problem is an external agent, be it the substance or the drug. From this it is anticipated that the possible outcomes may be failure (resumption of consumption) or a momentary result (to date I am still abstinent), as already found in others research (84, 85). The very reference to non-consumption keeps the user’s identity anchored to substances (by presence or absence), as highlighted by De Leon (86). The implicit transformation is from ‘active consumer’ to ‘abstinent consumer’, without contemplating an intervention that might lead one to consider other more personal and intentional aspects. The use of a psychologising vocabulary seems to serve several purposes, including diluting guilt and pandering to medicalising discourses, which are sometimes the most legitimized even among professionals. The terms ‘trauma’ and ‘demons’ take on justificatory value and an element of de-empowerment and passivity.

When treatment is seen as competence learning, community practices take on greater value in the eyes of the participants, as also reported by Mitchell et al. (87) who say that the pathway should be seen as a cyclical rather than linear accumulation of skills. Thus, greater compliance with community activities is promoted, helping them to read everyday activities as opportunities to work on themselves.

In this way, a greater dialogue is configured with the person accessing the service while maintaining all aspects of identity, both those linked to consumption and those that are not, as also highlighted by Best et al. (88), by De Maeyer (89) and by De Maeyer et al. (90). The representation of treatment as competence learning seems to allow the maintenance of a more integrated and situated identity. Starting from these assumptions, it seems fundamental to promote the development of the users’ perception of self-efficacy, to reinforce the perception of being able to actively determine their own change, which confirms the studies of Szulc (91).

In this configuration we find reference to a series of pragmatic goals (driving licence, job, medical check-ups) that are not present in other treatment configurations.

Configuring treatment as a generic place of life change leads participants to qualify the intervention as being useful when it helps to find a job, when it helps to acquire a driving licence or medical check-ups, and when it helps to find a home. Although these aspects may be of strategic importance, it is important to say that they cannot be confused with the purpose of the treatment.

While this favors a focus on the here and now, as well as on short-term goals, it also underlines how central it is to reflect on how users are involved in the design of interventions. This finding confirms the risk already pointed out by Neale et al. (92) about the possibility of a different point of view between service users and practitioners. In the absence of a shared representation of the treatment implemented, the user will not be able to configure the experience except within his or her previous experience. Indeed, this study confirms the importance of including the community user’s point of view and perception in the treatment setting, as also found by Goethals et al. (93). Within this framework we can ask ourselves the following questions. Is having an extremely vague idea of what will be proposed a potentiality or a criticality? How should this space of possibilities be exploited?

In fact, in most of the participants’ answers, goals and practices aimed at change often coincide: the aspiration to ‘get clean’ by divesting oneself of drugs and substances is simply pursued through its affirmation, without planning any strategy other than simply doing it in the ‘right’ ways. These experiences, without the necessary support for a re-signification of what has been done, will be placed within the user’s history as exceptions, limited experiences valid only within a specific context and at a specific time.

## 5 Conclusion and limits

As mentioned in the introduction, the therapeutic community model can combat drug use and abuse if the focus of intervention is the whole person and not just substances. For this reason, medication-assisted treatment (MAT) needs to be complemented with activities that allow new ideas of self and new identities to emerge. However, if people who use substances enter the community with substance use as the central problem, there is a risk that a common course of action cannot be shared.

The answers offered by the participants lead to a reflection on the implications of the total institution character of some residential services, particularly when combined with practices that bind the person to act only in the role of substance user/TC user or patient.

Conceiving of the TC as an isolated entity in which to ‘heal’ a person implies the assumption of individualistic theories on consumers and consumption, underestimating the interactive and contextual dimensions and the value of the multiplicity of social roles and interactive contexts that are experienced. Paradoxically, in these cases, while affirming the value of experience sharing, this is limited to TC members and the TC context, as if to assume a ‘salvific’ value of place. The implications of this approach can also be inferred from the way some participants offered a poor, bare and crushed representation of themselves in the role of addict and consumer. Blaming and infantilisation, risks inherent in any institution, need to be managed to prevent them from becoming obstacles to a process of taking responsibility and making self-care choices, as also noted by Chang et al. (94).

Our results strongly suggest the need to introduce moments of work aimed at building a work that is increasingly “with” the user and not “on” the user at all stages of the intervention.

Highlighting the possible implications of certain discourses, the need to integrate the various aspects involved in a coherent personal narrative is highlighted, being careful of the risks of medicalising the problem or limiting TC to a role of containing social discomfort. To this end, the introduction of narrative tools to accompany this process could be explored, to avoid the fragmentary nature of the experience that is characteristic of those who access institutions that are symbolically and spatially isolated.

Therefore, in the process of constructing a treatment approach that is “with” people and not “on” people, the requirement to produce a modification of the common-sense theories with which users interface with TCs when starting treatment emerges as a significant fact. As we have seen, many users enter the community believing that their main problem is detoxification and not the pathway that led to intoxication. This belief leads them to think that once they have achieved detoxification, they are cured. In fact, they ask to get out and then experience relapse.

They also have the theory that their problem is mainly psychological, delegating the resolution of any problem to psychology and using it to justify not changing (95). They also often theorize that they are saved by TC, again not focusing on their responsibility (96).

However, if, as noted above, these theories are the effect of the interaction between the users, the treatment institutions and all the voices within society concerning the theme of ‘addictions’, it is then evident that the representatives of the above-mentioned institutions (addiction services, national and local health systems, general practitioners, criminal justice operators) also need to be involved in order to generate such a modification (97). The implications that can be drawn from the research results allow us to anticipate that in the absence of a working pathway in this direction, shared with all agencies involved in combating substance use, the outcomes of interventions could be severely limited.

With regard to the limitations of the research, some participants were not accustomed to writing and narrating themselves through writing. Therefore, some of them struggled to report their thoughts. In some cases, the researcher reread and explained the questions present. The number of participants is sufficient to be able to explore the theories and discourses used by the users, but not yet sufficient to generalize the data, so we believe this work can be extended further.

This research did not divide the participants according to the type of addiction or even the type of substance used. For subsequent research, the interaction between the answers and the type of substance used could be tested. Another variable to consider for the future could be the number of years in the community, as some users experience more familiarity in the community than others and this could lead them to have a different view of treatment.

## Data Availability

The raw data supporting the conclusions of this article will be made available by the authors, without undue reservation.
